# In situ hybridization for the detection of rust fungi in paraffin embedded plant tissue sections

**DOI:** 10.1186/s13007-016-0137-3

**Published:** 2016-07-27

**Authors:** Mitchell A. Ellison, Michael B. McMahon, Morris R. Bonde, Cristi L. Palmer, Douglas G. Luster

**Affiliations:** 1 Department of Biology, University of Pittsburgh School of Medicine, Pittsburgh, PA USA; 2 USDA-ARS Foreign Disease-Weed Science Research Unit, Ft. Detrick, MD USA; 3 IR-4 Project, Rutgers University, Princeton, NJ USA

**Keywords:** Basidiomycota, Pucciniomycotina, Rust fungus, In situ hybridization, *Puccinia horiana*, *Uromyces transversalis*, *Phakopsora pachyrhizi*, *Chrysanthemum* × *morifolium*, *Gladiolus* × *hortulanus*, *Glycine max*

## Abstract

**Background:**

Rust fungi are obligate pathogens with multiple life stages often including different spore types and multiple plant hosts. While individual rust pathogens are often associated with specific plants, a wide range of plant species are infected with rust fungi. To study the interactions between these important pathogenic fungi and their host plants, one must be able to differentiate fungal tissue from plant tissue. This can be accomplished using the In situ hybridization (ISH) protocol described here.

**Results:**

To validate reproducibility using the ISH protocol, samples of *Chrysanthemum* × *morifolium* infected with *Puccinia horiana, Gladiolus* × *hortulanus* infected with *Uromyces transversalis* and *Glycine max* infected with *Phakopsora pachyrhizi* were tested alongside uninfected leaf tissue samples. The results of these tests show that this technique clearly distinguishes between rust pathogens and their respective host plant tissues.

**Conclusions:**

This ISH protocol is applicable to rust fungi and potentially other plant pathogenic fungi as well. It has been shown here that this protocol can be applied to pathogens from different genera of rust fungi with no background staining of plant tissue. We encourage the use of this protocol for the study of plant pathogenic fungi in paraffin embedded sections of host plant tissue.

## Background

Rust fungi (Basidiomycota, Pucciniomycotina) are obligate parasites that infect many species of vascular plants [1, 2]. Recent studies in this laboratory have focused on Chrysanthemum white rust, caused by *Puccinia horiana*, Gladiolus rust, caused by *Uromyces transversalis* and Asian soybean rust, caused by *Phakopsora pachyrhizi*. [3, 4]. Studies on the interactions between these pathogenic fungi and plants would benefit from approaches that allow visualization of the pathogen within host plant tissue, including the use of in situ hybridization (ISH) technology.

ISH was first used to localize specific DNA sequences on chromosomes using probes labeled with radioisotopes [5, 6]. The technique was later used for the detection of viral particles and high copy number mRNA in cultured cells and sectioned tissue making it useful for localizing gene expression patterns [6–10]. Non-radioactive methods were also developed that employed digoxygenin or biotin-conjugated nucleotides allowing for detection with antibody and streptavidin conjugates [9–11]. The development of non-radioactive methods eventually gave rise to fluorescent ISH (FISH), which employs various fluorescent-labeling techniques to produce fluorescence at the site of hybridization [12–14]. Non-radioactive ISH methods have been used to accomplish such tasks as chromosome mapping [14–16], gene expression localization [6, 8, 9, 11], and pathogen detection [17–26]. Chromogenic ISH (CISH) is an alternative to FISH that has become popular in diagnostic laboratories studying human pathogens [19–24].

Recently ISH has been used to identify microorganisms by targeting rRNA [18, 21, 24, 27–32]. The abundance of rRNA in the cell offers ample target for probes to bind to allowing for clear visualization of microorganisms within the sample being assayed. This technique has been used to identify and characterize prokaryotic organisms [27, 29, 32] and has been used for the detection of fungi in cultures [24, 31], plant [17, 18, 20, 30] and animal tissue [19, 21–23, 25].

The aim of this study was to develop a basic ISH protocol that plant pathologists can use for the detection of rust pathogens in paraffin embedded plant tissue. Here we report the development of an optimized protocol tested on three genera of rust fungi from three plant species. The results of this investigation demonstrate the utility of ISH as a tool for visualizing the infection of plant tissue by rust fungi.

## Methods

### Generation of infected leaf material

Leaves of *Chrysanthemum* × *morifolium* infected with *Puccinia horiana* isolate PA-11 [33], *Gladiolus* × *hortulanus* infected with *Uromyces transversalis* isolate CA-07 [34], and *Glycine max* infected with *Phakopsora pachyrhizi* isolate Taiwan 72-1 [35] were generated for experiments as described. These plant pathogens are regulated under the Plant Protection Act of 2000 and inoculations were conducted in a BSL-3 Plant Pathogen Containment Facility at Ft. Detrick MD under conditions specified in valid USDA APHIS PPQ 526 permits.

### DNA extraction and sequencing for ISH probe design

Genomic DNA was extracted from 50 mg of fungal basidiospores of *Puccinia horiana* using a hexadecyltrimethylammonium bromide (CTAB) extraction protocol beginning with 1 min of homogenization in 500 µL of CTAB extraction buffer (1 % CTAB, 0.7 M NaCl, 100 mM Tris (pH 7.5), 10 mM EDTA, 0.3 mg/mL proteinase K). Homogenized samples were incubated at 65 °C for 30 min, placed on ice for 2 min, and extracted with 500 µL of chloroform: isoamyl alcohol (24:1) by 10 s of vortexing followed by centrifugation at 14,000×*g* for 10 min. A volume of 300 µL of aqueous phase liquid was collected from each sample, combined with an equal volume of CTAB extraction buffer, re-extracted with 500 µL of chloroform: isoamyl alcohol (24:1), vortexed, and centrifuged at 14,000×*g* for 10 min. An equal volume of isopropanol was added to 400 µL of aqueous phase extract, which was gently mixed, and centrifuged at 14,000×*g* for 15 min. Following removal of isopropanol, DNA pellets were washed with 500 µL of 70 % ethanol, and centrifuged at 14,000×*g* for 20 min at 4 °C. Once 70 % ethanol was removed and nucleic acid pellets were allowed to dry a volume of 50 µL of TE buffer (10 mM Tris, pH 8.0, 1 mM EDTA) containing 1 mg/mL RNase A was added to each sample. Final concentrations of extracted DNA were determined using a Nanodrop 2000 (Thermo Fisher Scientific Inc, Waltham, MA).

Basidiospore DNA was amplified using primers for 18S rDNA (Table 2) in conventional PCR reactions and products were verified by gel electrophoresis. Post-amplification products were purified using ExoSAP-IT reagent (USB Corporation) before sequencing with BigDye Terminator version 3.1 cycle sequencing kit (ABI, Foster, CA). BigDye reaction products were purified using a DyeEx^®^ 2.0 Spin Kit (Qiagen), and analyzed on an ABI 3130XL sequencer (Applied Biosystems).

### Tissue sample collection and fixation

Rectangular leaf tissue samples measuring 4 cm by 1 cm were cut from infected leaves using a sterile razor blade and placed into a 50 mL conical tube containing 30 mL of FAE fixative (2 % formaldehyde, 5 % acetic acid, 60 % ethanol) (see supplementary protocol). After 48 h of incubation at 4 °C FAE fixative was removed from the sample tubes and samples were washed with 70 % ethanol for 5 min before incubation at room temperature in 70 % ethanol for one week. After fixation in FAE fixative and clearing in 70 % ethanol samples were shipped to American Histolabs (Rockville, MD) for RNase-free preparation, including paraffin embedding, sectioning, mounting on positively charged microscope slides, and deparaffinization. All microscope slide samples were stored at −80 °C prior to pre-hybridization.

### Prehybridization

All steps listed in the pre-hybridization protocol were carried out under ribonuclease (RNase) free conditions (see supplementary protocol). Slide mounted tissue samples were rehydrated by incubation in 100 % ethanol, 50 % ethanol, and diethylpyrocarbonate  (DEPC)-treated water for 3 min each. Following rehydration samples were treated with 0.2 M HCl for 20 min, washed for 2 min in DEPC-treated water, and incubated at 70 °C for 20 min in 2× SSPE (0.3 M NaCl, 2 mM EDTA, 20 mM NaH_2_PO_4_, pH 7.4), before digestion with 10ug/mL proteinase K in proteinase K buffer (20 mM Tris–HCl, pH 7.0, 2 mM CaCl_2_). Digestion was stopped by washing with 2× SSPE for 5 min at room temperature, and tissue was treated with 0.1 M TEA (0.1 M triethanolamine-Cl, pH 8.0) containing 0.5 % acetic anhydride (Sigma, St. Louis, MO) (v/v) for 10 min preceding treatment with biotin and streptavidin blocking solutions. Blocking of endogenous biotin is achieved by treating samples with 1 mL of 1× Blocking Reagent (Roche, Indianapolis, IN) containing 4 drops/mL of streptavidin blocker (Vector Laboratories, Burlingame, CA) for 15 min, and washing for 5 min in 2× SSPE before applying 1 mL of 1× Blocking Reagent containing 4 drops/mL of biotin blocker (Vector Laboratories, Burlingame, CA) for 15 min. After blocking, slides were washed in 2× SSPE for 5 min and dehydrated by incubation with DEPC-treated water, 50 % ethanol, and 100 % ethanol consecutively for 3 min each. Samples were allowed to air dry while hybridization mixture was prepared.

### Hybridization

Hybridization mixture (0.3 M NaCl, 20 mM Tris (pH 7.5), EDTA 2 mM, 500 µg/mL tRNA, 500 µg/mL poly(A) RNA, 1× Denhardt’s Solution [36], 10 % deionized formamide, 9 ng/µL DNA probe) was prepared and heated to 85–95 °C for 2 min before being placed into ice for 2 min. After the hybridization mixture was prepared, a volume of 100 µL was added to each sample slide, a coverslip was applied, and sample slides were placed into a plastic container lined with paper towels moistened with 4X SSPE. Sample slides were incubated overnight at 42 °C in one pint polypropylene snap-lid plastic containers in a H9270 Dual-Chamber Hybridization Oven (Thermo Fisher Scientific, Waltham, MA).

### Post hybridization, staining, and permanent mounting

Slides were removed from the 42 °C hybridization oven and dipped in 2× SSPE to aid in coverslip removal. Once coverslips were removed slides were washed in 2× SSPE for 30 min at room temperature, 1× SSPE for 30 min at 52 °C, and blocked for 30 min at room temperature using 1× Blocking Reagent. Samples were treated with Streptavidin-HRP at a concentration of 1:2500 (Invitrogen Carlsbad, CA.) in 1× PBS for 1 h at room temperature, and washed three times in 1× PBS, prior to application of ImmPACT™ VIP Peroxidase Substrate Kit (Vector Laboratories) following manufacturer’s instructions. To rinse and dehydrate tissue samples slides were placed consecutively into DEPC-treated water for 5 min, and 50 % ethanol and 100 % ethanol for 3 min each. Slides were allowed to dry completely before application of Permaslip Mounting Medium and Liquid Coverslip Solution (American MasterTech, Lodi, CA). Immediately following the addition of mounting media, a new coverslip was applied to permanently mount and preserve the tissue sections.

### Microscopy

All images were captured using a Nikon DS-Fi1 camera coupled with a Nikon Eclipse 80i microscope and NIS Elements imaging software (Nikon Inc., Melville, NY). The images reported here were produced through differential interference contrast (DIC) microscopy using a Nikon D-DA DIC light filter. A Nikon CFI Plan Achromat DL 10× objective lens was used to magnify samples to 100×.

## Results and discussion

**Fig. 1:**
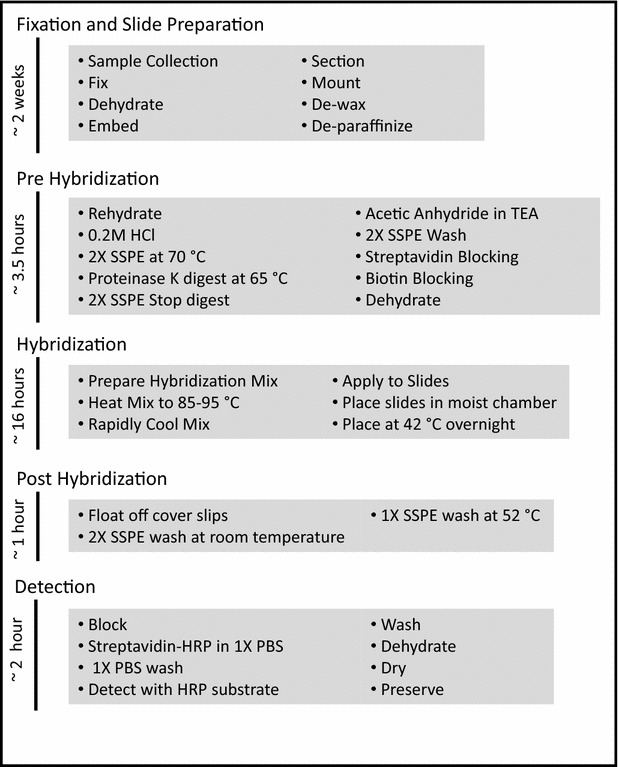
Simplified list of steps included in each phase of the *ISH* protocol with estimated length of time spent processing samples through each phase written vertically on the left hand edge

A review of the literature showed that very few studies had been conducted using ISH to target ribosomal RNA in a fungal pathogen during active infection [20, 30], several studies had targeted fungal 18S rRNA for ISH [18–24, 26, 30], and a few applied biotin-streptavidin as a reporter system [19, 37]. We believed that the field of plant pathology could benefit from the development of a non-radioactive ISH method designed for detecting rust fungi in host plant tissue. Therefore, a baseline protocol (Fig. 1) was developed from consensus information drawn from the current literature for further testing and refinement.

### Sequencing for probe design

Target sequences were required for development of probes to detect rust fungi in order to test the protocol. ISH probes for the targeted species were developed through a process of database searching, sequencing, and sequence alignment. The National Center for Biotechnology Information (NCBI) Taxonomy Browser website was used to search GenBank for 18S rRNA sequences from many fungal genera. Sequences (Table 1) were aligned in a CLUSTALW multiple sequence alignment using Biology Workbench 3.2 (http://workbench.sdsc.edu/). Sequencing primers were selected from highly conserved regions of the 18S sequence for the purpose of sequencing a variable region of the 18S gDNA of two rust fungi of interest to our laboratory; *Puccinia horiana* and *Uromyces transversalis*. These primers (Table 2) amplify a 596 bp fragment of the 18S rRNA gene. The newly obtained sequences were aligned to each of the sequences in Table 1 by pair wise local alignments, using NCBI Basic Local Alignment Search Tool (BLAST) (http://blast.ncbi.nlm.nih.gov/Blast.cgi). These alignments showed high variability when comparing rusts to non-rust fungi, but variation within rust fungi was low. From these alignments two 60 bp regions were chosen, and upon examining the properties of their sequences using OligoCalc (http://www.basic.northwestern.edu/biotools/oligocalc.html), a single region was selected. Probes were purchased from Integrated DNA Technologies (IDT) with biotin conjugated to their 5’ end for use ISH (probe sequences are listed in Table 2). The probes were designed to be exact matches to the 18S rRNA for their respective pathogen (Table 3). With precisely match probes, it was possible to refine the basic ISH protocol making modifications along the way based upon experimental results.

**Table 1:** Accession numbers of sequences used in CLUSTALW and BLAST alignments

Species	Accession
*Puccinia poarum*	GenBank:DQ831029
*Aspergillus sojae*	GenBank:D63696
*Aspergillus versicolor*	GenBank:AB008411
*Eurotium herbariorum*	GenBank:AB008402
*Cladosporium cladosporioides*	GenBank:AY251093
*Fusarium culmorum*	GenBank:AF548073
*Gremmeniella abietina*	GenBank:AF548074
*Monographella nivalis*	GenBank:AF064049
*Paecilomyces lilacinus*	GenBank:AB103380
*Penicillium brevicompactum*	GenBank:AF548083
*Rhizopus azygosporus*	GenBank:AB250156
*Stachybotrys sp.*	GenBank:DQ069246
*Trichoderma harzianum*	GenBank:AF548100
*Alternaria botrytis*	GenBank:AF548105
*Saccharomyces cerevisiae*	GenBank:Z75578
*Wallemia sebi*	GenBank:AF548108
*Phakopsora pachyrhizi*	GenBank:DQ354536

**Table 2:** List of sequencing primers and ISH probes used in this study

Primer/probe	Sequence
Primer 1 *Puccinia* 18S Forward (30 bp)	5′ CAATTGGAGGGCAAGTCTGGTGCCAGCAGC 3′
Primer 2 *Puccinia* 18S Reverse (30 bp)	5′ TGGACCTGGTGAGTTTCCCCGTGTTGAGTC 3′
*Puccinia horiana* 18S Anti-Sense	5′/biotin/AAGTTCACCAAGAGGTAAGCCTCCAACAA ATCAGTACACACCAAAAGGCAGACCAACTGC 3′
*Puccinia horiana* 18S Sense	5′/biotin/GCAGTTGGTCTGCCTTTTGGTGTGTACT GATTTGTTGGAGGCTTACCTCTTGGTGAACTT 3′
*Uromyces transversalis* 18S Anti-Sense	5′/biotin/AAGTTCACCAAGAGGTAAGCCTCCAACA AATCAGTACACACCAAAAGGCGGACCAACTGC 3′
*Uromyces transversalis* 18S Sense	5′/biotin/GCAGTTGGTCCGCCTTTTGGTGTGTACT GATTTGTTGGAGGCTTACCTCTTGGTGAACTT 3′
*Phakopsora pachyrhizi* 18S Anti-Sense	5′/biotin/AGGTTCACCAAGAGGTAAGCCTCCAAC AAATCAGTACACACCAAATGGCGGACCAACTGC 3′
*Phakopsora pachyrhizi* 18S Sense	5′/biotin/GCAGTTGGTCCGCCATTTGGTGTGTACT GATTTGTTGGAGGCTTACCTCTTGGTGAACCT 3′

**Table 3:** CLUSTALW alignment of anti-sense probes

Species	Sequence
*Uromyces transversalis*	AAGTTCACCAAGAGGTAAGCCTCCAACAAATCAGTACACACCAAAAGGCGGACCAACTGC
*Puccinia horiana*	AAGTTCACCAAGAGGTAAGCCTCCAACAAATCAGTACACACCAAAAGGCAGACCAACTGC
*Phakopsora pachyrhizi*	AGGTTCACCAAGAGGTAAGCCTCCAACAAATCAGTACACACCAAATGGCGGACCAACTGC
	* ******************************************* *** **********

### Development of an optimized ISH protocol

**Fig. 2:**
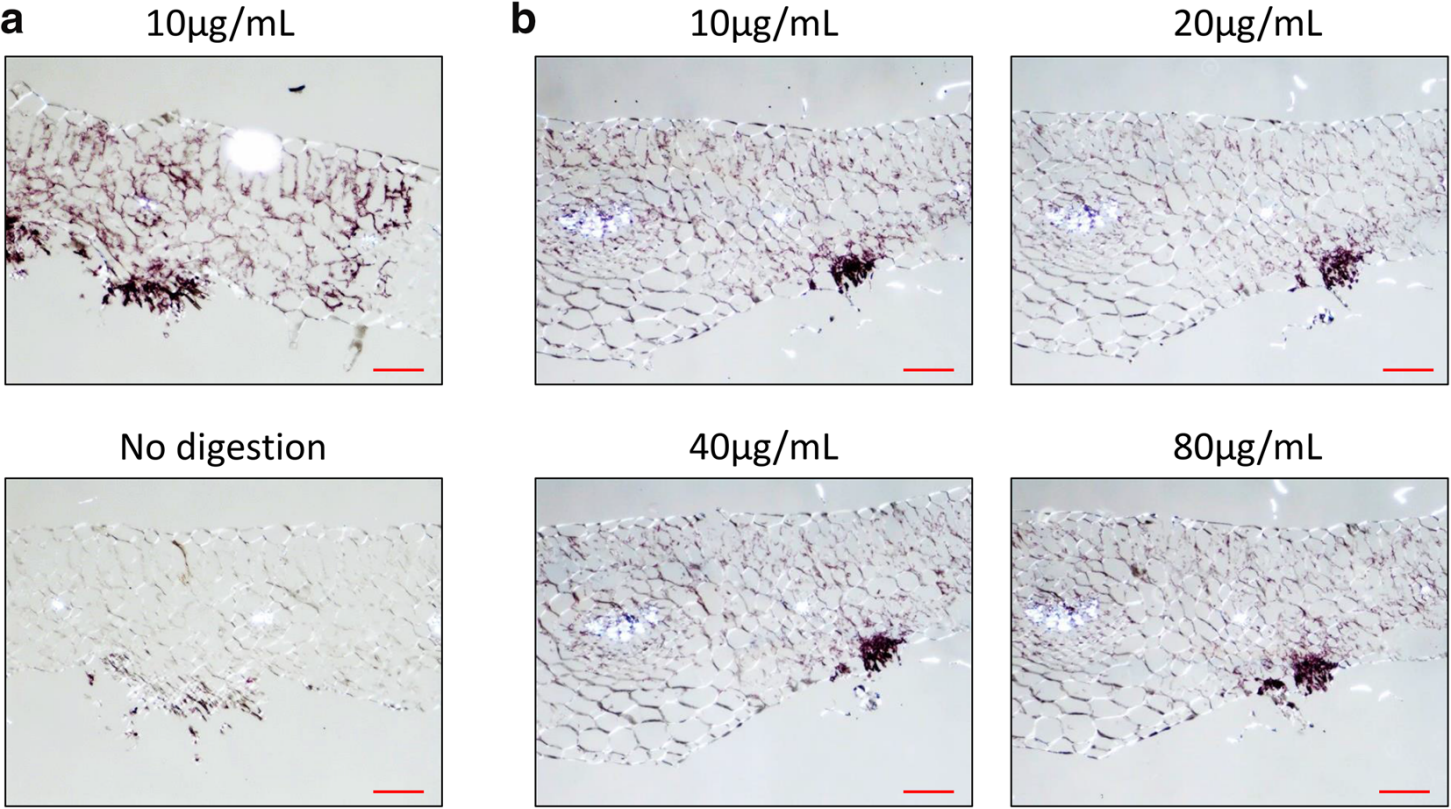
Images of samples of *C.* × *morifolium* infected with *P. horiana* prepared by ISH (*Red scale bar* = 100 μm). **a** Samples processed with (*top*) and without (*bottom*) a proteinase K digestion. **b** Samples processed using four different proteinase K concentrations. Purple staining of the tissue indicates hybridization signal generated by HRP reacting with purple substrate

**Fig. 3:**
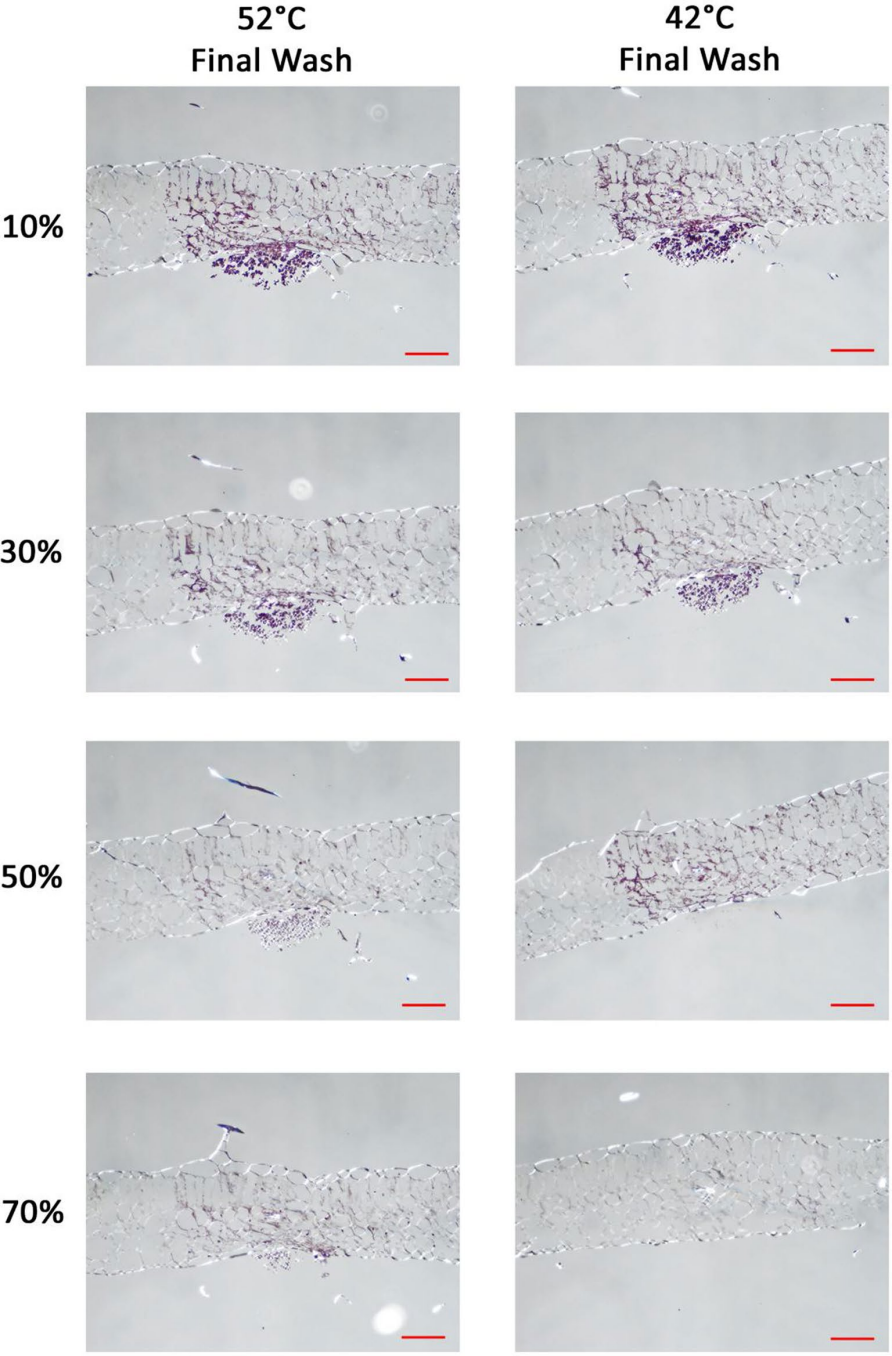
Images of *C.* × *morifolium* samples infected with *P. horiana* and prepared by ISH (*Red scale bar* = 100 μm). Photos are organized so that *columns* represent samples treated at a given final wash temperature (indicated by *column names*) and *rows* indicate samples treated with varying formamide concentrations (indicated by *row names*) in the hybridization mix for overnight hybridization. Purple staining of the tissue indicates hybridization signal generated by HRP reacting with purple substrate

**Fig. 4:**
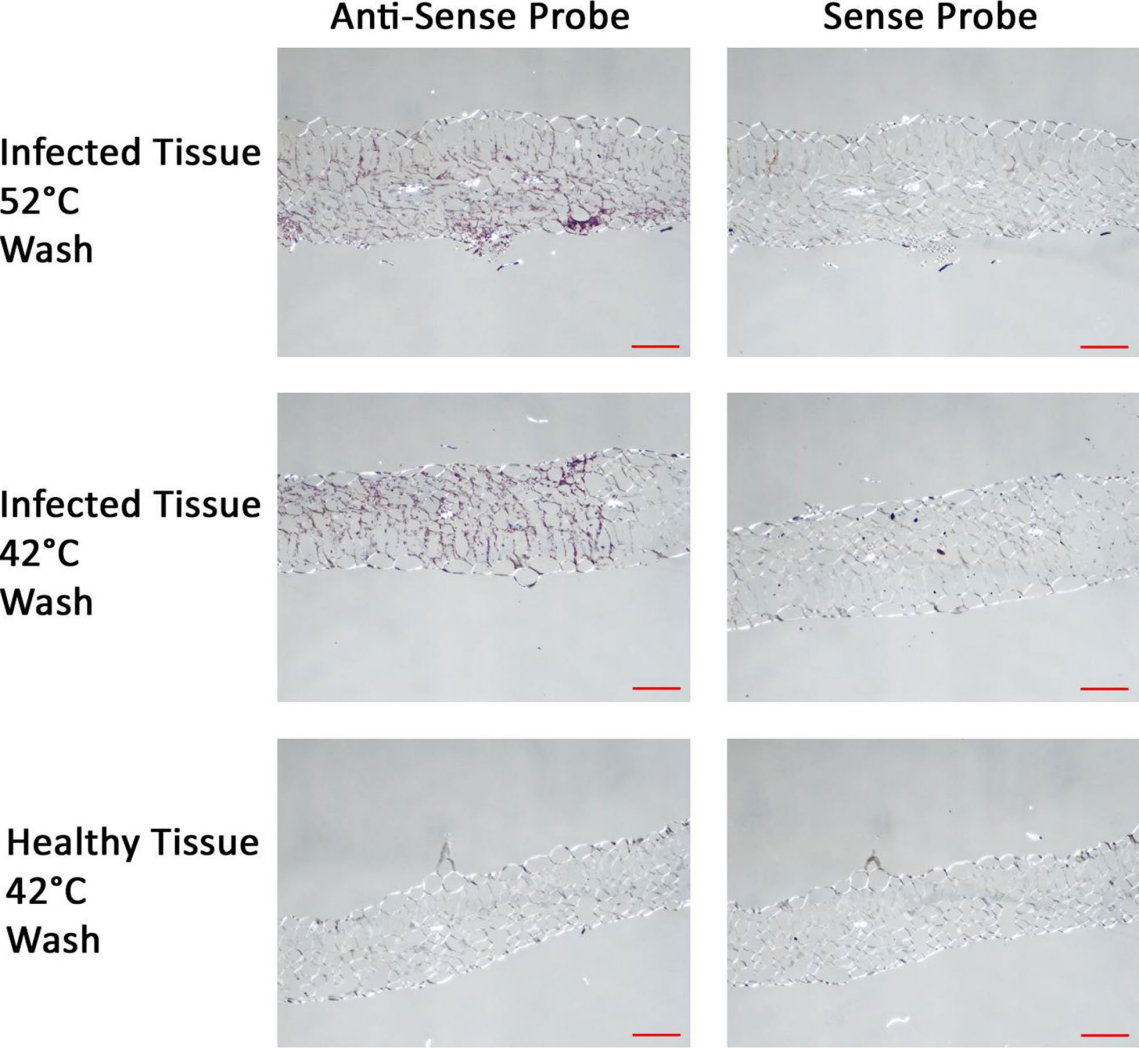
Images of *C.* × *morifolium* samples infected with *P. horiana* and prepared by ISH (*Red scale bar* = 100 μm). Photos are organized so that *columns* represent the probe used for hybridization (indicated by *column names*) and *rows* represent sample tissue type and post-hybridization wash temperature (indicated by *row names*). Purple staining of the tissue indicates hybridization signal generated by HRP reacting with purple substrate

We selected a few key steps in the ISH protocol to optimize the method, using *P. horiana*-infected chrysanthemum leaves as the test material. Steps tested were proteinase K concentration, presumably affecting accessibility of the target RNA within the tissue, formamide concentration, which controls the strength of the hybridization process, final wash temperature, which controls the stringency of hybridization, and probe concentration. The protocol was tested both with and without a proteinase K digestion (for 20 min with 10 µg/mL of proteinase K at 65 °C) and the results illustrated the necessity of this step, with no signal observed in the undigested samples (see Fig. 2a). Additionally, four proteinase K concentrations (10, 20, 40, and 80 µg/mL) were tested to determine an optimum concentration. A concentration of 10 µg/mL proteinase K worked best to allow probe access without disturbing tissue morphology (see Fig. 2b). Next four concentrations of formamide (10, 30, 50, and 70 %) were tested along with two wash temperatures (42 and 52 °C carried out in 1× SSPE for 30 min). Minimal differences were observed between wash temperatures, but formamide concentrations showed large differences, with lower concentrations resulting in increased signal (see Fig. 3). The results of this experiment led to the selection of two formamide concentrations for testing (50 and 10 %) with healthy plant control samples and sense probe (which should not hybridize to the target RNA sequence). Negligible background was observed in the sense-probe-treated infected tissue with a 42 °C wash and no background was observed in healthy plant samples treated with either probe. The optimal signal was obtained with 10 % formamide in the hybridization mix and the least background was observed with a 52 °C wash (see Fig. 4). Lastly probe concentration was varied (1, 3, and 9 ng/µL), and increased signal was observed when using 9 ng/µL with no additional background. These empirically determined conditions discovered as the result of these experiments were incorporated into a final optimized protocol. The protocol had, at this point, only been tested using sections of *Chrysanthemum* × *morifolium* infected with *Puccinia horiana* and further testing was required to determine the generality and reproducibility of the protocol.

### Application of the protocol to rust fungi

**Fig. 5:**
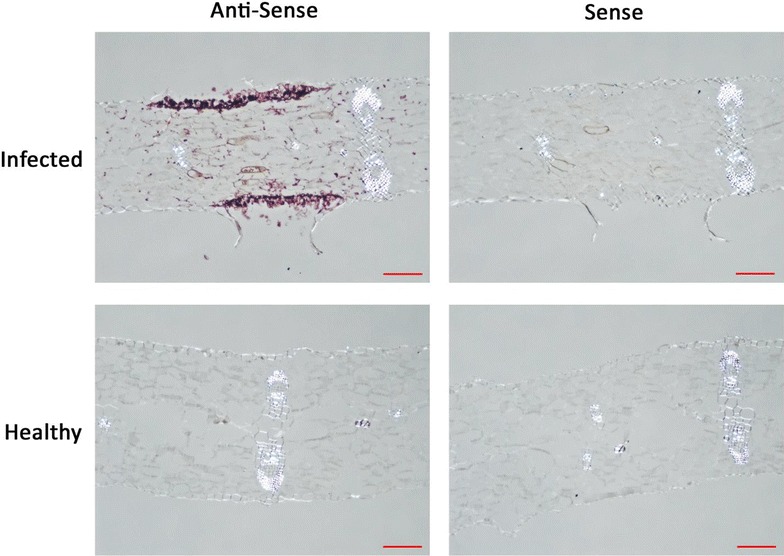
Images of *G.* × *hortulanus* samples infected with *U. transversalis* and prepared by ISH (*Red scale bar* = 100 μm). Photos are organized so that *columns* represent the probe used for hybridization (indicated by *column names*) and *rows* represent sample tissue types (indicated by *row names*). Purple staining of the tissue indicates hybridization signal generated by HRP reacting with purple substrate

**Fig. 6:**
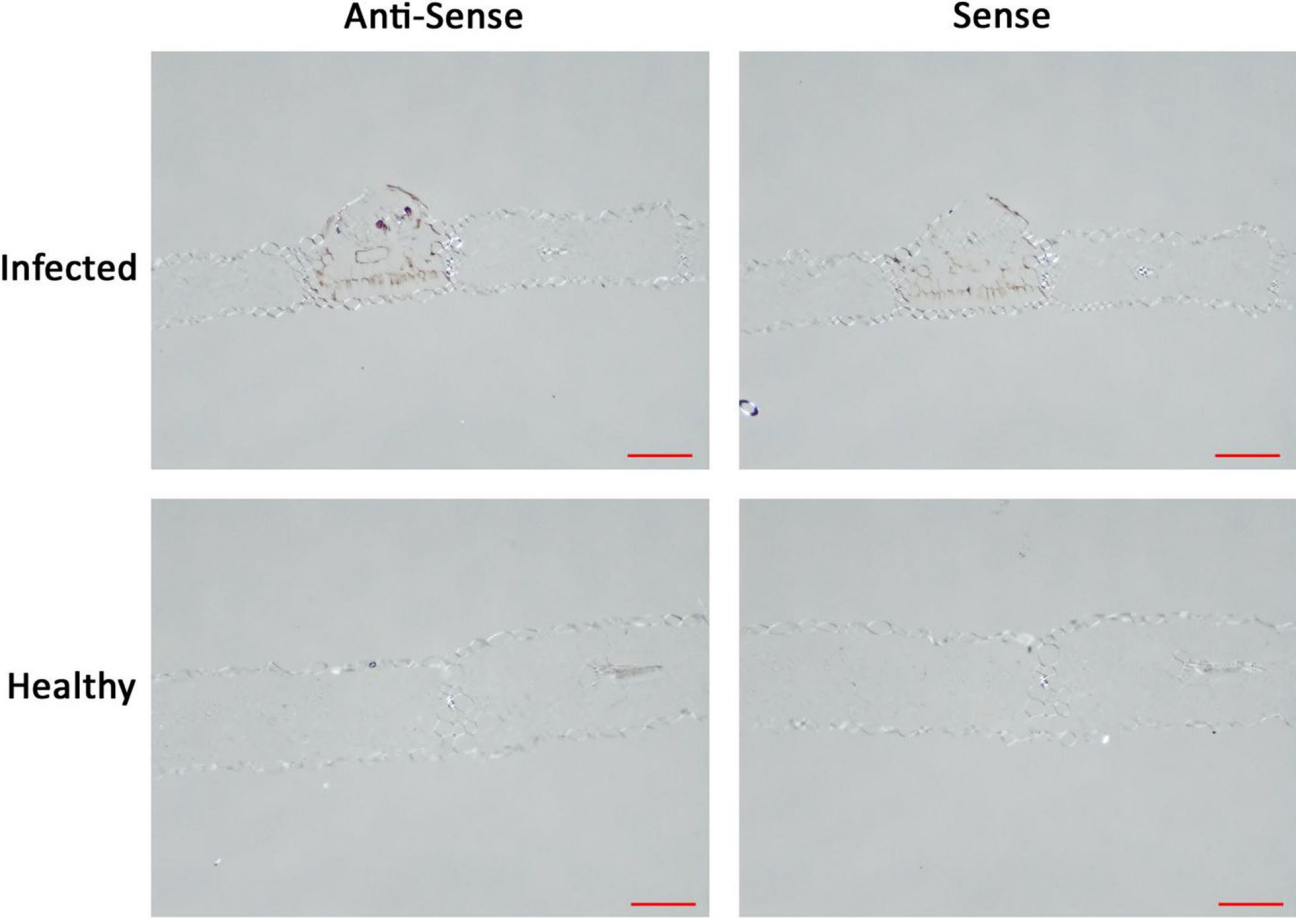
Images of *G.* *max* samples infected with *P. pachyrhizi* and prepared by ISH (*Red scale bar* = 100 μm). Photos are organized so that *columns* represent the probe used for hybridization (indicated by *column names*) and *rows* represent sample tissue types (indicated by *row names*). Purple staining of the tissue indicates hybridization signal generated by HRP reacting with purple substrate

In order to validate the applicability and reproducibility of the refined protocol, samples were taken from two other research subjects in our laboratory; *Gladiolus* × *hortulanus* infected with *U. transversalis* and *Glycine max* infected with *P. pachyrhizi*. Slides containing samples of both pathogens were treated with both sense and anti-sense probes using the optimized protocol. These experiments demonstrated that the refined ISH protocol is effective on species within the genera Puccinia (Figs. 2, 3, 4) Uromyces and Phakopsora (Figs. 5, 6). When applied to *Glycine max* infected with *P. pachyrhizi*, the signal was weak compared to the other two species (Fig. 6). *U. transversalis* samples showed strong signal in all slides prepared with the anti-sense probe and no signal in the infected tissue prepared with the sense probe. Healthy plant tissue processed with both sense and anti-sense probes showed no signal, demonstrating further that this technique is preferentially staining pathogen tissue and not the host plant. The strength of signal obtained from *U. transversalis* samples was equivalent to signal observed in *P. horiana* samples. *P. pachyrhizi* showed weak signal in one out of five slides prepared with anti-sense probe and the other four appear the same as slides treated with the sense probe. The fact that some signal was observed indicates that this technique requires further optimization for *P. pachyrhizi*. In particular the alteration of the high temperature steps could potentially increase signal strength by reducing sample loss.

The images presented here are at relatively low magnification, and resolution is therefore at the tissue level. Further magnification of images did not resolve at the intracellular level (not shown). Use of a fluorescent label rather than precipitable stain may result in higher-level resolution.

## Conclusion

We present here a generalized ISH protocol for localization of rust fungi in paraffin embedded sections of host tissues. This technique provides plant pathologists with a tool to study the morphology of rust fungi within the plants they infect, which may aid in the elucidation of the life cycles of these plant pathogenic fungi and determination of fungal growth patterns in host tissue. We have demonstrated that this protocol can be applied to pathogens from different genera of rust fungi residing in different plant hosts with little to no non-specific background staining of plant tissue. Our protocol is easy to apply and modify if necessary. The generic protocol thus serves as a starting point that may be modified to suit other plant pathogen systems of interest.

## References

[1] Dean R, Van Kan JA, Pretorius ZA, Hammond-Kosack KE, Di Pietro A, Spanu PD, Rudd JJ, Dickman M, Kahmann R, Ellis J (2012). The Top 10 fungal pathogens in molecular plant pathology. Mol Plant Pathol..

[2] Yamaoka Y (2014). Recent outbreaks of rust diseases and the importance of basic biological research for controlling rusts. J Gen Plant Pathol.

[3] Bromfield KR (1984). Soybean Rust (Monograph).

[4] Schneider RW, Hollier CA, Whitam HK (2005). First report of soybean rust caused by *Phakopsora pachyrhizi* in the continental United States. Plant Dis.

[5] Gall JG, Pardue ML (1971). Nucleic acid hybridization in cytological preparations. Methods Enzymol.

[6] Zeller R, Rogers M, Haramis AG. *In situ* hybridization to cellular RNA. Curr Protoc Mol Biol. 2001:14.13. 11–14.13. 16. 10.1002/0471142727.mb1403s55 18265111

[7] Brahic M, Haase AT (1978). Detection of viral sequences of low reiteration frequency by *in situ* hybridization. Proc Natl Acad Sci USA.

[8] Haramis A, Carrasco A. Whole-mount *in situ* hybridization and detection of RNAs in vertebrate embryos and isolated organs. Curr Protoc Mol Biol. 1996:14.19. 10.1002/0471142727.mb1409s66 18265339

[9] Lawrence JB, Singer RH (1985). Quantitative analysis of *in situ* hybridization methods for the detection of actin gene expression. Nucleic Acids Res.

[10] Singer R, Lawrence JB, Villnave C (1986). Optimization of *in situ* hybridization using isotopic and nonisotopic detection methods. Biotechniques.

[11] Singer RH, Ward DC (1982). Actin gene expression visualized in chicken muscle tissue culture by using *in situ* hybridization with a biotinated nucleotide analog. Proc Natl Acad Sci USA.

[12] Landegent J, De Wal NJI, Baan R, Hoeijmakers J, Van der Ploeg M (1984). 2-Acetylaminofluorene-modified probes for the indirect hybridocytochemical detection of specific nucleic acid sequences. Exp Cell Res.

[13] Pinkel D, Landegent J, Collins C, Fuscoe J, Segraves R, Lucas J, Gray J (1988). Fluorescence *in situ* hybridization with human chromosome-specific libraries: detection of trisomy 21 and translocations of chromosome 4. Proc Natl Acad Sci USA.

[14] Pinkel D, Straume T, Gray J (1986). Cytogenetic analysis using quantitative, high-sensitivity, fluorescence hybridization. Proc Natl Acad Sci USA.

[15] Brandriff B, Gordon L, Fertitta A, Olsen A, Christensen M, Ashworth L, Nelson D, Carrano A, Mohrenweiser H (1994). Human chromosome 19p: a fluorescence *in situ* hybridization map with genomic distance estimates for 79 intervals spanning 20 Mb. Genomics.

[16] Gordon L, Bergmann A, Christensen M, Danganan L, Lee D, Ashworth L, Nelson D, Olsen A, Mohrenweiser H, Carrano A (1995). A 30-Mb metric fluorescence *in situ* hybridization map of human chromosome 19q. Genomics.

[17] Assmus B, Hutzler P, Kirchhof G, Amann R, Lawrence JR, Hartmann A (1995). *In situ* localization of azospirillum brasilense in the rhizosphere of wheat with fluorescently labeled, rRNA-targeted oligonucleotide probes and scanning confocal laser microscopy. Appl Environ Microbiol.

[18] Glöckner FO, Amann R, Alfreider A, Pernthaler J, Psenner R, Trebesius K, Schleifer K-H (1996). An *in situ* hybridization protocol for detection and identification of planktonic bacteria. Syst Appl Microbiol.

[19] Li AY, Crone M, Adams PJ, Fenwick SG, Hardy GE, Williams N (2014). The microscopic examination of *Phytophthora cinnamomi* in plant tissues using fluorescent *in situ* hybridization. J Phytopathol.

[20] Montone KT, Guarner J (2013). *In situ* hybridization for rRNA sequences in anatomic pathology specimens, applications for fungal pathogen detection: a review. Adv Anat Pathol..

[21] Montone KT, LiVolsi VA, Lanza DC, Kennedy DW, Palmer J, Chiu AG, Feldman MD, Loevner LA, Nachamkin I (2011). *In situ* hybridization for specific fungal organisms in acute invasive fungal rhinosinusitis. Am J Clin Pathol.

[22] Schröder S, Hain M, Sterflinger K (2010). Colorimetric *in situ* hybridization (CISH) with digoxigenin-labeled oligonucleotide probes in autofluorescent hyphomycetes. Int Microbiol..

[23] Shinozaki M, Okubo Y, Sasai D, Nakayama H, Ishiwatari T, Murayama S, Tochigi N, Wakayama M, Nemoto T, Shibuya K (2012). Development and evaluation of nucleic acid-based techniques for an auxiliary diagnosis of invasive fungal infections in formalin-fixed and paraffin-embedded (FFPE) tissues. Med Mycol.

[24] Sterflinger K, Hain M (1999). *In situ* hybridization with rRNA targeted probes as a new tool for the detection of black yeasts and meristematic fungi. Stud Mycol.

[25] Tanaka E (2009). Specific *in situ* visualization of the pathogenic endophytic fungus Aciculosporium take, the cause of witches’ broom in bamboo. Appl Environ Microbiol.

[26] Vági P, Knapp DG, Kósa A, Seress D, Horváth ÁN, Kovács GM (2014). Simultaneous specific *in planta* visualization of root-colonizing fungi using fluorescence *in situ* hybridization (FISH). Mycorrhiza.

[27] Amann R, Fuchs BM, Behrens S (2001). The identification of microorganisms by fluorescence in situ hybridisation. Curr Opin Biotechnol.

[28] Bloch B (1993). Biotinylated probes for *in situ* hybridization histochemistry: use for mRNA detection. J Histochem Cytochem.

[29] Brigati DJ, Myerson D, Leary JJ, Spalholz B, Travis SZ, Fong CK, Hsiung G, Ward DC (1983). Detection of viral genomes in cultured cells and paraffin-embedded tissue sections using biotin-labeled hybridization probes. Virology.

[30] Moter A, Göbel UB (2000). Fluorescence *in situ* hybridization (FISH) for direct visualization of microorganisms. J Microbiol Methods.

[31] Wagner M, Horn M, Daims H (2003). Fluorescence *in situ* hybridisation for the identification and characterisation of prokaryotes. Curr Opin Microbiol.

[32] Zachgo S, Gilmartin PM, Bowler C (2002). In situ hybridization. Molecular plant biology.

[33] Bonde MR, Palmer CL, Luster DG, Nester SE, Revell JM, Berner DK (2013). Sporulation capacity and longevity of Puccinia horiana teliospores in infected chrysanthemum leaves. Plant Health Prog..

[34] Bonde MR, Nester SE, Luster DG, Palmer CL (2015). Longevity of *Uromyces transversalis,* causal agent of gladiolus rust, under various environmental conditions. Plant Health Prog.

[35] Lamour KH, Finley L, Snover-Clift KL, Stack JP, Pierzynski J, Hammerschmidt R, Jacobs JL, Byrne JM, Harmon PF, Vitoreli AM (2006). Early detection of Asian soybean rust using PCR. Plant Health Prog.

[36] Denhardt DT (1966). A membrane-filter technique for the detection of complementary DNA. Biochem Biophys Res Commun..

[37] McManus J, Cason JE (1950). Carbohydrate histochemistry studied by acetylation techniques: I. Periodic acid methods. J Exp Med..

